# Radioactivity Levels and Heavy Metal Concentration in Mining Areas in Zacatecas, Mexico

**DOI:** 10.3390/toxics12110818

**Published:** 2024-11-14

**Authors:** Edmundo Escareño-Juarez, Rocío Fernández-Saavedra, M. Belén Gómez-Mancebo, Ana I. Barrado, Ana I. Cardona, Isabel Rucandio

**Affiliations:** 1 Unidad Académica de Estudios Nucleares, Universidad Autónoma de Zacatecas, Calle Ciprés No. 10, La Peñuela, Zacatecas 98000, Mexico; 2 División de Química, Centro de Investigaciones Energéticas, Medioambientales y Tecnológicas (CIEMAT), Avda. Complutense 40, 28040 Madrid, Spain; rocio.fernandez@ciemat.es (R.F.-S.); mariabelen.gomez@ciemat.es (M.B.G.-M.); anaisabel.cardona@ciemat.es (A.I.C.); isabel.rucandio@ciemat.es (I.R.); 3 Dosimetría de Radiaciones Ionizantes, Centro de Investigaciones Energéticas, Medioambientales y Tecnológicas (CIEMAT), Avda. Complutense 40, 28040 Madrid, Spain; anaisabel.barrado@ciemat.es

**Keywords:** soil contamination, Zacatecas, Mexico, level of radioactivity, heavy metals

## Abstract

The state of Zacatecas (Mexico) is one of the places most affected by pollution from the mining industry. A total of 21 samples from two areas were collected (6 samples from zone A and 15 samples from zone B) to evaluate the level of radioactivity and the degree of contamination with heavy metals. The activity concentration for ^238^U and ^232^Th was within typical values but that of ^226^Ra exceeded the value of 60 Bq kg^−1^ in both areas. In some places, the concentration of ^40^K was higher than the world average according to UNSCEAR. The radium equivalent activity Ra_eq_ and outdoor gamma exposure dose (D) indicated that some sites presented a radiological risk for the population. The excess lifetime cancer risk (ELCR) presented a higher risk than the world average in both sampled areas. Other parameters, such as the radiation index R_i_, the external risk index H_ex_, and the internal risk index H_in_, also indicated that there was a greater risk due to radiation exposure in these areas. In the case of heavy metals, some parameters, such as the EF, the I_geo_, and the CF, showed that some places in zone A and zone B were contaminated with As and Pb. However, the concentration of selenium obtained by ICP-MS in the sampled soil was higher than the average in the Earth’s crust, both in mine waste dumps and in unaltered soil, which indicated that the background concentration for selenium in these places is higher than the world average.

## 1. Introduction

Radioactivity and heavy metal content in soil have been investigated by many authors because the human health effects induced by the exposure to these chemicals are known [1]. Although natural radioactivity typically remains at low and nonharmful levels, specific geological formations may exhibit higher concentrations, resulting in increased natural background radiation. Moreover, human activities, including mining, industrial processes, and the improper disposal of radioactive materials, can substantially elevate environmental radioactivity levels [2]. So, in soils, during the study of radioactivity, the issue of high levels of bioavailable heavy metals (HMs) should be addressed, as recommended by the International Atomic Energy Agency technical report 419 [3], since they can have both technological and regulatory implications. These issues are of high interest in mining areas. In this context, this present paper obtained data regarding the radioactivity and total metal content of the silver mines located in Zacatecas (México). This study provides the basis on which measures can be taken to alleviate pollution due to mining activities in the area.

Human exposure to radiation is primarily due to natural sources: cosmic radiation and the terrestrial radionuclides present in the environment since the formation of the Earth [1,4]. The natural radioactivity of the soil is mainly due to the presence of the parent series ^238^U, ^232^Th, and ^40^K [5,6]. The specific terrestrial radiation levels depend on geological and geographic conditions. On the other hand, the most important artificial radionuclide is ^137^Cs, which is found in the soil because of anthropogenic activities, that is, tests and accidents with nuclear weapons in the atmosphere, and represents a persistent environmental pollutant due to its long half-life (T_1/2_ = 30.1 years) and its strong affinity for binding to the soil [1]. Studies on soil radioactivity can offer essential baseline data for evaluating the exposure of local populations to external gamma-ray irradiation and internal irradiation resulting from the transfer of radionuclides from the soil to plants consumed in the human diet [7]. Moreover, the concentrations of ^226^Ra and ^232^Th in the soil directly affect the levels of ^222^Rn and ^220^Rn gases, which are alpha emitters present in the air. These radioactive gases and their short-lived decay products pose significant risks to human lung tissues and constitute the primary source of internal radiation exposure [8]. Prolonged exposure to high levels of radiation can result in health problems such as cancer, genetic mutations, and other chronic diseases. Additionally, radiation can affect ecosystems, influencing biodiversity and the ecological balance. Understanding the distribution and concentration of radionuclides in various environmental matrices is important for assessing potential risks and implementing effective mitigation strategies [2].

HMs are present in the environment at natural concentrations. The main natural sources of HMs in soils are volcanic eruptions and the weathering of metal-rich rocks [9]. However, the levels of HMs present in the environment have increased considerably during the last decades mainly due to human activity [10]. Among the anthropogenic activities that are considered sources of toxic metals in the environment are industry in general, atmospheric deposition, agriculture (fertilizers, pesticides, and amendments), waste and landfills, and mining activities. Contamination from the mining industry represents an important problem in many countries since it induces serious harmful impacts on the health of the local environment due to the release of HMs, which are the most dangerous anthropogenic contaminants due to their toxicity and persistence in nature [11]. The presence of HMs in agricultural soils raises significant environmental and public health concerns. Mining activities, especially when appropriate treatment measures are not implemented, can lead to the substantial heavy metal contamination of mining sites. This contamination may subsequently migrate to the surrounding farmlands. Numerous case studies have documented elevated levels of heavy metal contamination in agricultural soils near mining areas, with concentrations sometimes reaching hundreds of times higher than those found in unpolluted soils [12]. When discussing toxicity, it is important to consider not just the total concentration of the element but also its bioavailability, meaning the amount available for uptake by living organisms. Prolonged exposure to heavy metals (HMs) is linked to chronic health conditions. The soil–plant–human pathway is a significant environmental route through which humans can ingest radionuclides and HMs [13].

There are several processes that govern metals’ behavior in the soil, which interact dynamically, making the prediction of the fate and effects of metals in the environment a complex task. Processes such as adsorption, absorption, redox reactions, parent rock alteration, and the acidification buffering capacity are influenced by biogeochemical factors such as the pH value, the cation exchange capacity (CEC), the redox potential, the element in question, and its chemical speciation: elements that can exist in various oxidation states or chemical compounds [14].

As previously stated, mining is considered one of the most important sources of HMs. During the last decades, most of the attention has focused on pollution from large-scale mining. However, the environmental pollution related to artisanal and small-scale mining has been neglected. Most established companies have substantially improved their production techniques and taken responsibility for the remediation of contaminated land to meet increasingly stringent regulatory requirements and due to public pressure [15]. Mining activities are prone to releasing toxic elements into soils through tailings, worthless ores, and subsequent smelting operations, which have had unfavorable consequences for human health and aquatic environments [16]. The residues of old mines have a strong impact and pose a threat to the environment, aquatic biota, and health because of outdated extraction methods and their high contents of HMs such as As, Pb, Cd, Hg, Cu, Zn, and Fe, which can cause a significant damage [17]. HMs can enter the food chain through agriculture, surface waters, animal feed, industrial emissions, and waste. Crops, animals, and their derived products can become contaminated due to soil, water, or air affected by these metals [18,19]. Selenium (Se) is an essential trace element that is a component of the amino acids selenocysteine and selenomethionine. However, at elevated levels, selenium can be toxic, and instances of toxicity and mortality have been documented following acute exposure to supraphysiological concentrations or to certain chemical forms. Anthropogenic activities such as mining are the main sources of Se contamination [20,21,22].

The soil acts as a physical, chemical, and biological filter against contamination and is a purifying agent that acts as a sink for contaminants. The soil, then, plays a role in the environment by cleaning the surface and underground waters as well as our crops and ultimately our food and that of all living organisms. As an important part of the terrestrial ecosystem, soil is the most basic natural resource for humans, and it can be constantly regenerated and recycled. The rational utilization of soil resources and the protection of the soil environment are worldwide concerns [23]. In this sense, the objective of this work was to determine the radioactivity and heavy metal content in soil samples collected from mining areas in the state of Zacatecas, Mexico, as an example of artisanal and small-scale mining and its impact on the environment.

## 2. Materials and Methods

### 2.1. Study Area

**Figure 1:**
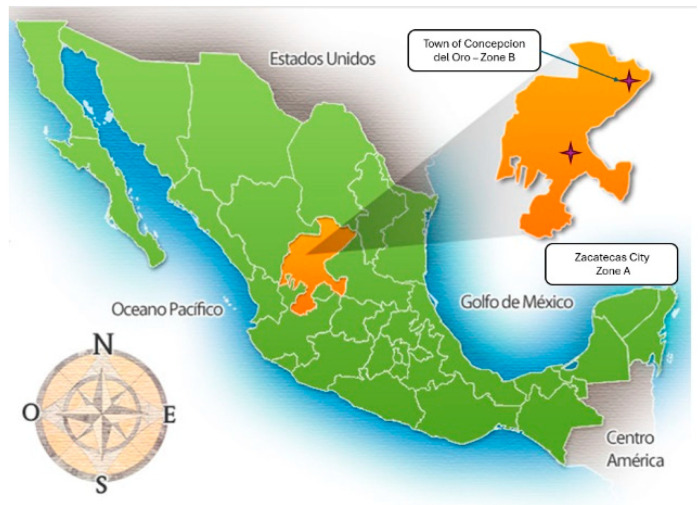
Map of the Mexican Republic and the location of the state of Zacatecas (https://mr.travelbymexico.com/759-estado-de-zacatecas/ accessed on 7 November 2024).

Mexico is one of the countries most affected by soil contamination with HMs, especially the state of Zacatecas, which is also the most important silver producer in the country [24]. With an area of 77,684 km^2^, Zacatecas (Figure 1) is the eighth largest state of the 32 states of the Mexican Republic.

Three-quarters of the territory of Zacatecas correspond to arid and semiarid zones. Among these, 14% offers beneficial conditions for agriculture, 79% for livestock, and 7% is covered by timber and nontimber forests. In the central part of the state, there are chestnut soils, abundant in semiarid zones and characterized by a layer of caliche or loose lime. This type of soil is promising for the development of agriculture and livestock. In the northeast of Zacatecas, there are soils characteristic of the arid and semiarid zones of central and northern Mexico [25].

**Figure 2:**
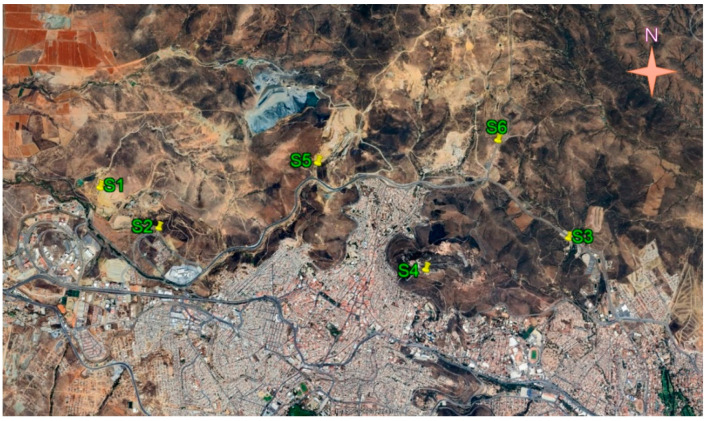
Locations of the sampling places in zone A near the city of Zacatecas (Google Earth Pro 7.3.6.9796 (64-bit)).

**Figure 3:**
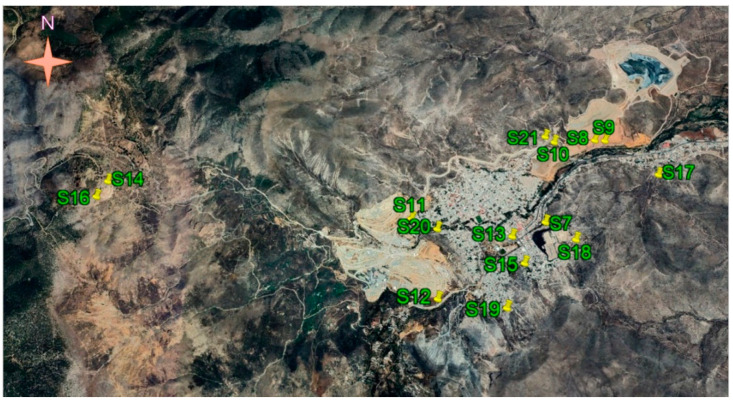
Locations of the sampling places in zone B near the city of Concepcion del Oro (Google Earth Pro 7.3.6.9796 (64-bit)).

In this work, two mining areas of the state of Zacatecas were sampled. Zone A (Figure 2) was located on the outskirts of Zacatecas city, the state capital. Zone B (Figure 3) was located 260 km northeast of the capital in the municipality of Concepción del Oro. Both areas had a mining history of at least 200 years. In each area, two kinds of samples were collected: (a) mine tailing soils and (b) undisturbed soils. The sampled areas were selected based on their proximity to residential zones and/or protected areas. Samples were collected from a surface area of one square meter to a maximum depth of 10 cm. Subsequently, grinding, homogenization, and sieving processes were performed on the samples. In total, 21 samples were collected during July and August 2021, transferred to the laboratory, and dried at 50 °C for five days. A total of 2 kg of soil was collected from each site for the various analyses. All samples were homogenized and sieved at 2 microns. The official Mexican standard NOM-141-SEMARNAT-2003 [26] defines tailings as solid waste generated in primary mineral separation and concentration operations.

Table 1 shows the sampling location and types of samples for both zone A and zone B.

### 2.2. Radioactivity Analysis

Radioactivity was measured in the laboratory of the Unidad Académica de Estudios Nucleares de la Universidad Autónoma de Zacatecas, México. For radioactivity determination, ~500 g was weighed and allowed to sit for 30 days in a sealed Marinelli container to allow ^226^Ra and its progeny to reach equilibrium.

The activity concentrations of the samples were measured using a high-purity germanium (HPGe) detector with a relative efficiency of 38%. The energy resolution of the HPGe detector was 1.8 keV at a 1.33 MeV ^60^Co gamma line. A 16K multichannel analyzer was used to obtain the spectrum, and the analysis was performed using GENIE-2k v. 3.4 software (Canberra Industry). The samples were measured for 80,000 s (approx. 22.2 h) with an operating voltage of 3900 volts. The system was previously calibrated for energy and efficiency using calibrated sources.

Considering the complexity of the samples, the gamma-radiation-emitting radionuclides analyzed in this work were conveniently grouped. The grouping of radionuclides proposed by Murray et al. [27] and Gil-Pacheco et al. [28] was used; the classification of radionuclides used in this work is shown in Table 2.

In group A, only ^238^U was considered, and the gamma-emitting radionuclides selected were ^234^Th and ^234m^Pa. ^230^Th, belonging to group B, was not considered in this work due to its low concentration. Group C included ^226^Ra and ^222^Rn and their progeny; the activity concentration of ^226^Ra was determined from the γ peak at 186 keV by suppressing the gamma interference of the ^235^U peak at 185.7 keV. The activity concentrations of ^214^Pb and ^214^Bi were determined by measuring the γ energies at 351 and 609 keV, and 1120 keV and 1764.5 keV, respectively. ^210^Pb from group D and ^235^U from group E were not used for this work either due to their low concentrations in nature or because others of greater importance interfere with some photopics of these radionuclides. The critical element of group F is ^228^Ac, and the γ peak at 911 keV was selected to determine its activity concentration. The ^232^Th activity concentration of group G was averaged with the γ rays at 238.6 keV and 583.2 keV emitted by ^212^Pb and ^208^Tl, respectively.

In addition to the natural gamma-emitting radionuclides of the ^238^U, ^235^U, and ^232^Th series, other natural and artificial gamma emitters were analyzed, such as ^40^K and ^137^Cs, which were measured using the photopics at 1460.83 and 661 keV, respectively.

Different parameters and radiological hazard indices were calculated from the data obtained from the radiation measurements of the samples: radio equivalent activity (Ra_eq_) [13], absorbed dose ($\dot{D}$) [28,29], effective dose (E) [28], excess lifetime cancer risk due to gamma-ray radiation (ELCR) [1], external risk index (H_ex_) [13], internal risk index (H_in_) [13], and the radioactivity index (R_i_) [28].

The *radio equivalent activity* (${Ra}_{eq}$) describes the gamma emission of different naturally occurring radionuclides in a material and was calculated using the following formula:

(1) $${Ra}_{eq}=C_{Ra}+1.43C_{Th}+0.077C_{K}$$

where $C_{Ra}$, $C_{Th}$, and $C_{K}$ are specific activities of ^226^Ra, ^232^Th, and ^40^K, respectively. The recommended limit of *Ra*_*eq*_, given by UNSCEAR 2000, is 370 kg^−1^ [5,30].

The *absorbed dose* ($\dot{D}$) of gamma radiation at 1 m above the ground surface was determined using the following formula:

(2) $$\dot{D}=0.0417C_{K}+0.462C_{Ra}+0.604C_{Th}$$

where $C_{K}$, $C_{Ra}$, and $C_{Th}$ are, respectively, the activity mass concentrations (in Bq kg^−1^) of ^40^K, ^226^Ra, and ^232^Th in nGy h^−1^. The global average indoor absorbed gamma dose rate is 57 nGy h^−1^ [30].

The absorbed dose rate in the air due to the measured activity concentration values of the different groups (*DP*, nGy h^−1^) in the samples under study was converted into the *effective dose rate* (E), in mSv y^−1^, using the following expression:

(3) $$E=\dot{D}*O*C*8760*10^{-6}$$

where O (equal to 0.2) is the outdoor occupancy factor, and C (equal to 0.7 Sv Gy^−1^) is the conversion factor from the dose absorbed in the air to the effective dose received by an adult person. World average value of 0.46 mSv y^−1^ [30].

The *ELCR* [27] due to gamma-ray radiation per 100,000 people was estimated using the following expression:

(4) $$ELCR=E*DL*RF*10^{-3}$$

where E is the annual effective dose in mSv, DL is the lifespan (equal to 70 years), and RF is the risk of fatal cancer per Sv (equal to 0.057 Sv^−1^). The *ELCR* is used to characterize the elevated risk of cancer due to chronic exposure to gamma rays. The global average ELCR is 1.45 × 10⁻^3^ [30].

$H_{ex}$ provides an assessment of the health risk associated with the emission of gamma radiation by various natural radionuclides, while $H_{in}$ estimates the internal exposure of living cells to radon and its progeny [13].

$H_{ex}$ and $H_{in}$ were calculated as follows:

(5) $$H_{ex}=\frac{C_{Ra}}{370}+\frac{C_{Th}}{259}+\frac{C_{K}}{4810}$$

(6) $$H_{in}=\frac{C_{Ra}}{185}+\frac{C_{Th}}{259}+\frac{C_{K}}{4810}$$

where $C_{Ra}$, $C_{Th}$, and $C_{K}$ are the specific activities of ^226^Ra, ^232^Th, and ^40^K, respectively. The limit of these indices should be less or equal to unity, as reported by the UNSCEAR and ICRP [31,32].

The *R_i_* proposed by Gil-Pacheco et al. [28] was calculated using the following formula:

(7) $$R_{i}=\frac{C_{GA}}{30,000}+\frac{C_{GC}}{375}+\frac{C_{GE}}{4200}+\frac{C_{GF}}{800}+\frac{C_{GG}}{450}+\frac{C_{K}}{4200}+\frac{C_{Cs}}{1400}$$

where C_GX_ is the activity concentration of group X (where X is the values of groups A, C, E, F, and G), and C_K_ and C_Cs_ are the activity concentrations of K and Cs, respectively.

### 2.3. Chemical and Structural Characterization

Fifty grams of each sample was transported to the Centro de Investigaciones Energéticas, Medioambientales y Tecnológicas (CIEMAT), Spain, for chemical and structural characterization.

Major elements were determined by wavelength-dispersive X-ray fluorescence (WDXRF) with an AXIOS Spectrometer (Malvern-PANalytical—Almelo, The Netherlands), in pressed powder pellets previously prepared in an automatic press (HTP-40, Herzog, Osnabrück, Germany). The pellets were analyzed with a semiquantitative method developed by Malvern-PANalytical.

The previous acid digestion of the samples in a temperature-controlled microwave oven was required using a Milestone Ethos 1 device to put the sample into solution for the following determinations.

Sodium and potassium were determined by flame atomic emission spectroscopy (FAES) in a Perkin-Elmer (Shelton, CT, USA) 2280. Minor and trace elements were determined by inductively coupled plasma optical emission spectroscopy (ICP-OES) (Agilent Technologies Spain, S.L., Barcelona, Spain) and inductively coupled plasma mass spectrometry (ICP-MS) (Thermo Scientific, Darmstadt, Germany) as a function of the element concentrations: ICP-OES was used for concentrations higher than 0.05 mg/L and ICP-MS for lower concentrations.

ICP-OES measurements were performed by using a benchtop dual-view ICP-OES with a vertical torch, Agilent 5110 model (Agilent Technologies Spain, S.L., Spain). Two or three different wavelengths were used for each element quantification, and the averages of each element line were considered in cases of noninterference.

ICP-MS analyses were conducted with a Thermo iCAP-RQ spectrometer (Thermo Scientific, Darmstadt, Germany) equipped with a quadrupole mass analyzer and an electron multiplier detector. A Meinhard nebulizer (Meinhard, CO, USA) with a baffled cyclonic spray chamber and a peristaltic pump were used for sample introduction.

The crystalline phases were identified by X-ray diffraction (XRD) with an X’Pert Pro diffractometer (Malvern-PANalytical) with Cu Ka radiation (λ = 1.54 Å) operating at 45 kV and 40 mA. XRD data were collected in θ–θ configuration in the angular range of 5 < 2θ < 80 with a 0.017 step size.

### 2.4. pH in Water and CaCl_2_

The pH determination was carried out according to Technical Norm ASTM-D4972-13 [33]. The pH measurements were carried out in water and a calcium chloride solution (CaCl_2_ 0.01 M) because calcium displaces part of the exchangeable aluminum. The low ionic strength counteracts the dilution effect on the exchange equilibrium by setting the salt concentration of the solution closer to that expected in soil solution. The pH values obtained in the calcium chloride solution are slightly lower than those measured in water due to the release of more aluminum ions, which are then hydrolyzed. Therefore, both measurements are required to entirely define soil pH. The measurement was made with an Orion model 550 pH meter, using an Orion 8102BN electrode. The pH meter was calibrated at 3 points (4, 7 m and 10) using certified buffer solutions from Mettler-Toledo. Soil samples were dried and sieved to 2 μm. Two solutions were prepared in a 1:1 liquid-to-solid ratio: 10 mL of water/10 g of soil and 10 mL of 0.01 M CaCl_2_/10 g of soil. Both solutions were left under stirring for 2 h and allowed to rest for one hour; then, the pH of each of the solutions was measured to obtain the soil’s pH value. Each solution was measured twice.

### 2.5. Parameters of Impact of HMs

To measure the degree of impact of the HMs in the sampled soils, three parameters were determined.

*Enrichment factors* (*EFs*)*:* EFs are used to evaluate the degree of heavy metal contamination. The EF is estimated by using the following equation:

(8) $$EF=\frac{{\left(\frac{C_{i}}{C_{ref}}\right)}_{Sample}}{{\left(\frac{C_{i}}{C_{ref}}\right)}_{Background}}$$

where $C_{i}$ is the concentration of the target element, and $C_{ref}$ is the concentration of the reference element [34]. Iron was chosen as the reference element due to its low coefficient of variation (CV) in these samples.

The baseline metal concentrations from the standard preindustrial reference level (in mg kg^−1^) [34] considered in this work were 15 for As, 1.0 for Cd, 90 for Cr, 68 for Ni, 70 for Pb, 0.6 for Se, 0.9 for Tl, 2.7 for U, and 10.5 for Th. EF classification is as follows: EF < 1 indicates no enrichment, EF < 3 is minor enrichment, EF = 3–5 is moderate enrichment, EF = 5–10 is moderately severe enrichment, EF = 10–25 is severe enrichment, EF = 25–50 is very severe enrichment and EF > 50 is extremely severe enrichment [34].

*The geoaccumulation index*$(I_{geo}$) [34]: I_geo_ is applied to quantify metal contamination in soils and is calculated using the following equation:

(9) $$I_{geo}={log}_{2}\left[\frac{C_{n}}{1.5B_{n}}\right]$$

where $C_{n}$ is the metal concentration measured in the soil samples in a study area, $B_{n}$ is the background value of the corresponding metal (the values mentioned for the previous parameter), and 1.5 is the background matrix correction due to lithological effects. I_geo_ is categorized into seven degrees or classes of contamination: (i) $I_{geo}$ > 5 = extremely contaminated, (ii) $I_{geo}$ = 4–5 = heavily to extremely contaminated, (iii) $I_{geo}$ = 3–4 = heavily contaminated, (iv) $I_{geo}$ = 2–3 = moderately to heavily contaminated, (v) $I_{geo}$ = 1–2 = +moderately contaminated, (vi) $I_{geo}$ = 0–1= not contaminated to moderately contaminated, and (vii) $I_{geo}$ < 0 = not contaminated [34].

*The contamination factor* (*CF*)*:* The CF is the relationship obtained by dividing the concentration of each metal in the soil ($C_{Hm})$ by the base or background value (concentration in uncontaminated soil ($C_{Bk}$)) [34]:

(10) $$CF=\frac{C_{Hm}}{C_{Bk}}$$

Pollution levels can be classified according to their intensities on a scale ranging from 1 to 6 (0 = none, 1 = none to medium, 2 = moderate, 3 = moderately to strongly contaminated, 4 = heavily contaminated, 5 = strongly to very strong, 6 = very strong) [34].

## 3. Results and Discussion

### 3.1. Radioactivity Levels and Their Impact

The activity concentrations of natural and human-made radionuclides in mine tailings and undisturbed soils were evaluated. The radioactivity evaluation was carried out in order to estimate various parameters, such as the absorbed dose and the probability of cancer risk due to continuous exposure to ionizing radiation as a result of the ingestion and inhalation of radionuclides from the soil matrix collected in the study area.

The results obtained in the gamma spectrometry system of zone A and B samples are shown in Table 3.

The specific activity of ^238^U varied from 51.22 to 160.18 Bq kg^−1^ in the 21 samples collected in the two studied areas. For ^226^Ra, a range between 105.76 and 596.26 Bq kg^−1^ was found, ^232^Th varied from 9.66 to 58.93 Bq kg^−1^, and the specific activity concentration for ^228^Th ranged between 6.70 and 69.26 Bq kg^−1^. ^40^K ranged from 324.17 to 1356.02 Bq kg^−1^. The anthropogenic radioisotope of ^137^Cs ranged between 0.07 and 6.35 Bq kg^−1^.

Table 3 also presents the range and mean of the global activity concentrations of ^40^K, ^238^U, ^226^Ra, and ^232^Th according to UNSCEAR 2000 [5]. Comparing these values with those found in zones A and B, it was observed that there was a high concentration of ^40^K activity in zone A. Unlike zone B, some sampled zones presented low concentrations of ^40^K, and others had high concentrations.

In the case of ^238^U (group A), almost all the activity concentrations were above the UNSCEAR global range, as did that of ^226^Ra (group C). However, ^232^Th (group F) was in the world range reported by UNSCEAR.

More recent specific activity data are shown in the UNSCEAR 2008 report, Annex B, page 233, paragraph 77 [35]. Annex C The current global average values are 33 Bq kg^−1^ for ^238^U, 32 Bq kg^−1^ for ^226^Ra, and 45 Bq kg^−1^ for ^232^Th. The mean value for ^40^K, 412 Bq kg^−1^, is also close to the previous value (400 Bq kg^−1^). Although the average natural concentrations of radionuclides in soils are low, there is wide variation, with reported levels of up to 1000 Bq kg^−1^ for ^238^U, 360 Bq kg^−1^ for ^232^Th, and 3200 Bq kg^−1^ for ^40^K [35].

The activity concentration of ^40^K was widely distributed across the study area. The highest activity concentration of the natural radionuclides in the soil was obtained from sample S6 in zone A; this area is characterized by limestone. This study revealed a nonuniform distribution of these radionuclides, which is due to the geological variability in the area.

Table 4 shows the average specific activity concentrations in various places worldwide. It was concluded that the four main natural radionuclides had very variable activity concentrations from one place to another. In our samples, ^238^U, ^226^Ra, and ^40^K were found in high concentrations compared with the averages shown in this table, but ^232^Th was within the average in this table.

### 3.2. Radionuclide Contamination Assessment 

The activity concentrations of ^226^Ra, ^232^Th, and ^40^K in the soil samples varied significantly. The radius equivalent (Ra_eq_) was used to address this issue. Ra_eq_ represents the activity of a sample in terms of an equivalent amount of ^226^Ra [36]. This assumes that all radiation from the other radionuclides in the sample can be attributed to the presence of ^226^Ra. The formula for Ra_eq_ considers the specific activities of ^226^Ra, ^232^Th, and ^40^K in a soil sample. Ra_eq_ provides a more straightforward way of comparing the radiation levels of different soil samples despite their nonuniform distribution of radionuclides [37].

The average Ra_eq_ value of the soil samples in shown Table 5 was, for many samples, above the internationally recommended limit of 370 Bq [37], and there were sites with double this value. Thus, we conclude that they pose a risk to the population in some places.

Most samples from zones A and B had an outdoor gamma exposure dose value (D) above 100 nGy h^−1^. According to UNSCEAR, the average outdoor gamma exposure dose is calculated as 54 nGy h^−1^ [35].

All samples from both areas presented an effective dose ratio (E) below or within the normal range according to UNSCEAR [35] from 0.3 to 1.0 mSv y^−1^. The average worldwide exposure to natural radiation sources is 2.4, ranging from 1 to 10 mSv y^−1^ (UNSCEAR 2000) [4]. According to the values obtained from the annual effective dose from natural radionuclides shown in Table 5, the exposure is low for the population.

Long-term exposure to radiation can pose a risk of causing cancer. This implies that every individual has a risk of cancer at a time in their lifetime. The world’s average ELCR per 100,000 people is 2.9 × 10^−4^ [13], and, in Table 5, the ELCR values are above this value in all the studied samples.

**Figure 4:**
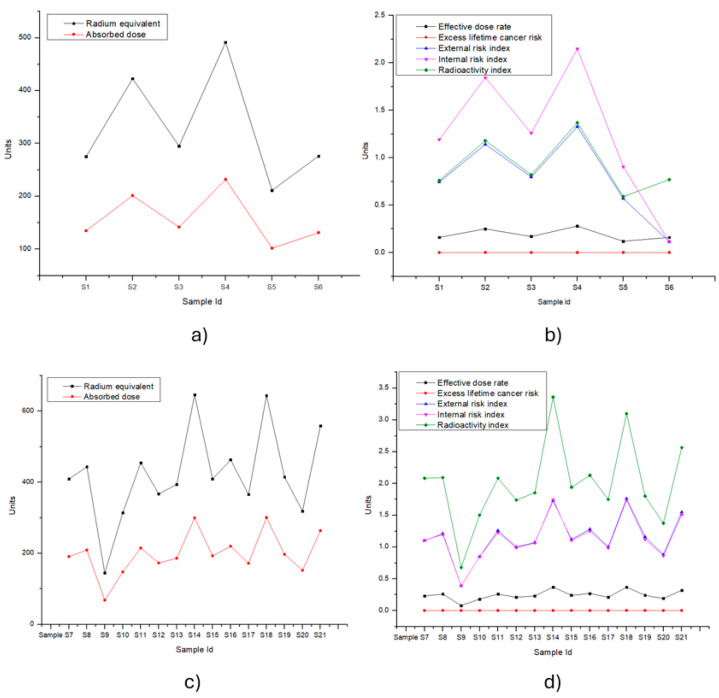
The values of the parameters obtained from zones A and B. The units for the graphed parameters are radius equivalent Bq kg^−1^, absorbed dose nGy h^−1^, effective dose ratio mSv y^−1^, lifetime excess cancer risk mSv, external risk mSv y^−1^, internal risk mSv y^−1^, radioactivity index nGy h^−1^. Graphs (**a**,**b**) correspond to the data obtained from the six samples in zone A. Graphs (**c**,**d**) are the results of the 14 samples from zone B.

Figure 4 shows the values of the radiological parameters obtained in the two sampled areas to visualize the results presented in Table 5.

The radiation index Ri was close to one in all samples from zone A, but, in zone B, the value was slightly higher than one, which implies that there was a somewhat higher risk of irradiation in zone B.

The average of *H_ex_* in zone A was less than one, but there were two samples that exceeded one, and the average *H_ex_* in zone B exceeded the value of one. The average of *H_in_* exceeded a value of one in zone A, while the average of *H_in_* in zone B was equal to two.

### 3.3. Results of the Chemical and Structural Characterization

The results of the XRF chemical analysis of the major elements are shown in Table 6. In the sampling points located in zone A, the major elements are Si, Fe, Al, and Ca, which are elements normally found in soils with average values corresponding to 20%, 6.5%, 6.2% and 3.8%, respectively. K and S are also found in high concentrations with average values of 1.6% and 1.5%, respectively. These elements are not normally found in such high concentration in soils.

The results for zone B (Table 6) also showed Si, Fe, Ca and Al concentrations associated with the major elements found in common soils. However, in this case, the Ca concentration was higher than the Si concentration, which is normally the major element in natural soils. Calcareous soils often contain more than 15% CaCO_3_, which can occur in different forms (powders, nodules, crusts, etc.). Soils with a high CaCO_3_ content belong to the WRB Calcisols group and related calcareous subgroups. They are found in arid areas, such as a part of the territory of the state of Zacatecas. Calcareous soils often suffer from micronutrient deficiencies, especially zinc and iron. It can be seen from Table 6 that as the Ca content increases, the Fe content decreases. In this case, high concentrations of S and K were also found.

Table 7 show the results obtained by the ICP-OES and ICP-MS techniques in mg kg^−1^ for the HMs present in the collected samples.

According to the official Mexican standard NOM-147-SEMARNAT/SSA-2004 [38] in Table 7, the average concentration of As wis higher than that permitted for soil for residential use, but for soil for industrial use, the average concentration of As is within the allowed values. According to this official standard, the average concentrations of Cd, Cr, Ni, Pb, Se, and Tl are within the values permitted for residential use.

Table 8 shows the crystalline phases identified in zone A. The compound common to all of them was SiO_2_ (mostly quartz). In some of the samples, Fe_2_O_3_ and CaCO_3_ were also present. The silicates present were those normally found in association with quartz, i.e., albite, muscovite, and orthoclase. The other compounds found were CaF_2_ in sample S1 and CaSO_4_ in sample S6.

The crystalline phases identified in zone B are also shown in Table 8. In this case, in most of the samples, CaCO_3_ was present, corroborating the XRF results. This phase is representative of arid soils such as those found in this sampling zone. SiO_2_ and Fe_2_O_3_ phases are typically found in soil samples. Some of the samples contained CaSO_4_ (anhydrite), which could be natural or added to the soil (as gypsum). CaSO_4_ addition is considered a good initial procedure to prepare nonarable soil, as it allows the permeability of the soil to last for several years, helping increase the penetration of fertilizers applied on the surface. The silicate phases present are those normally found in association with quartz, i.e., albite, muscovite, and orthoclase, as in zone A.

### 3.4. Heavy Metal Contamination Assessment 

The EFs are summarized in Table 9. Only samples S1 and S16 to S21 had severe As enrichment (EF = 10–25) and S2 for Pb. A large number of samples (S2, S5, S7, S9, S10, and S12) had moderately severe As enrichment (EF = 5–10). The other elements also presented moderately severe enrichment: S1, S17–S19, S21 for Cd; S1 for Pb; S3, S7, S16–S21 for Se; S17 and S21 for Tl. It is also worth noting the samples that had moderate enrichment (EF = 3–5): S3 and S14 for As; S20 for Cd; S1, S5, S11, and S12 for Se.

The most critical element is **As**. The EFs for As indicated that all samples were enriched in As, from minor to severe enrichment (S1 in zone A and S16–S21 in zone B). It is clear that this element has a naturally high concentration, and given its ease of mobilization, it is enriched in many areas due to the anthropogenic activity. Only sample S1 from zone A presented a moderate to severe EF for **Cd**; however, in zone B, samples S17–S21 also presented a moderate to severe EF for Cd. These last samples were from undisturbed soil, except for sample 21, which was mine waste. This finding indicates that Cd was present in high natural concentrations in zone B. **Cr** enrichment was minor in sample S6, belonging to zone A, but it does not represent a risk. **Ni**, **U**, and **Th** had a lower EF, so it was concluded that there was no enrichment of these elements in either area. In the case of **Pb**, samples S1 and S2 from zone A had moderate to severe EFs, both samples being mine waste. However, in zone B, all the samples had no enrichment or minor enrichment of Pb. With respect to **Se**, only the EFs for S4 and S15 indicated no enrichment; the rest had EFs ranging from minor to moderately severe enrichment in both zones. It could be concluded that, as in the case of As, the area had high concentrations of Se. In fact, sample S3, which was the most enriched sample in zone A, was from undisturbed soil. In zone B, among the samples with moderately severe enrichment were S16 to 20, which were undisturbed soils, and S7 and S21, which were from mine tailings. Finally, there were only two samples, S17 and S21, from zone B, that had a moderate to severe EF for **Tl**.

The results of the *I_geo_* are shown in Table 10. Samples S1, S2, S3, and S5 from zone A and almost all samples from zone B were moderately to heavily contaminated with **As**. No samples from either areas presented a significant level of geoaccumulation of **Cd**, **Ni**, **Tl**, **U**, or **Th**. In addition, in Table 10, it can be observed that **Pb** contamination was moderate to heavy in samples S1 and S2 from zone A and in S7 from zone B, and moderate in S15 and S16. In addition, almost all samples from zones A and B show moderate to heavy **Se** contamination.

The CF results are presented in Table 11. For this parameter, it was observed that there was high **As** contamination in both areas. Only some samples presented a CF of less than three, which is a moderate to no contamination level. The **Cd** contamination was limited. Only some samples, such as S1 from zone A and S17 to S21 from zone B, presented substantial Cd contamination. Samples S1 and S2 from zone A and samples S7, S15, and S16 showed extreme to strong **Pb** contamination. Most samples from zones A and B had very strong **Se** contamination; only sample S4 from zone A did not show Se contamination. Sample S2 from zone A and samples S17 and S21 from zone B presented moderately strong to extremely strong **Tl** contamination. In zone A, no **U** contamination was found, but, in zone B, sample S7 had moderate contamination. No significant contamination was found for the rest of the HMs analyzed.

## 4. Conclusions

The concentrations of radionuclides and HMs were measured in the soils from two mining areas in Zacatecas, Mexico. The specific activity concentrations for ^238^U in zones A (near the city of Zacatecas) and B (near the city of Concepcion del Oro) were within the ranges proposed by UNSCEAR but were much higher than the world average. The concentration of ^226^Ra activity was very high in both areas, exceeding the maximum value (60 Bq kg^−1^) recommended by UNSCEAR [5]. Several samples from both areas had a concentration above the maximum limit for ^40^K, and the total average was also high. The activity concentration of ^232^Th was within the UNSCEAR range of 11 to 64 Bq kg^−1^.

The recommended limit for radium equivalent activity Ra_eq_ is 370 Bq; sites that exceeded that value were found, indicating a risk to the population in some places. The absorbed dose ($\dot{D}$) value of 54 nGy h^−1^ was exceeded for almost all samples. The effective dose rate (E) was within the normal range according to UNSCEAR [35]. The *ELCR* presented a higher risk than the world average in both sampled areas. The average *R_i_*, the average *H_ex_*, and the average *H_in_* exceeded a value of one, presenting a greater risk to human health due to radiation.

The major components found in all samples were Si, Fe, Al, and Ca, but, in zone B, the concentration of Ca, which is normally the major element in natural soils, was higher than that of Si. This finding is characteristic of the calcareous soils present in arid areas, such as part of the territory of Zacatecas.

According to the official Mexican standard NOM-147-SEMARNAT/SSA-2004, the average concentration of As in soils for residential use was higher than allowed, and the average concentration of Pb was within the permitted limits in both zones.

Some areas had severe enrichment factors for As and Pb. Some sites showed anthropogenic As enrichment. Se was found in high concentrations in both undisturbed soil and mine tailings in both areas. Cd was also present in some samples from both zones but especially in zone B, which indicated that the natural Cd concentration was high. Cr, Ni, U, and Th had low enrichment factors.

The $I_{geo}$ indicated that some samples from zone A and almost all samples from zone B had moderate to heavy As contamination. Pb only strongly geoaccumulated in two samples from zone A and one sample from zone B. This index for Se indicated moderate to heavy contamination in almost all samples from both areas. Important contamination with Cd, Ni, Tl, U, and Th was not found in any sample.

Considering the contamination factor, it was observed that both areas were highly contaminated with As. Most samples from zones A and B had very strong Se contamination (high EF, Igeo, and CF values); this agrees with the Se being found in high natural concentrations in this region of Mexico. Some samples from both areas presented strong contamination with Pb. Excessive contamination was not found for the other analyzed elements.

This study evaluated the concentrations of radionuclides and HMs to propose future solutions for mitigate their effects, which will benefit the population and ecosystems.

## Figures and Tables

**Table 1:** Locations of zone A and B samples.

	Sample	Type of Sample	Location
Zone A	S1	Mine tailings	22°47′15″ N 102°36.36″ W
	S2	Mine tailings	24°36′48″ N 101°24′51″ W
	S3	Undisturbed soil	22°46′52″ N 102°32′42″ W
	S4	Undisturbed soil	22°46′36″ N 102°33′52″ W
	S5	Mine dump	22°47′25″ N 102°34′46″ W
	S6	Undisturbed soil	22°79′31″ N 102°55′53″ W
Zone B	S7	Mine tailings	24°37′12″ N 101°24′40″ W
	S8	Mine tailings	24°37′16″ N 101°24′30″ W
	S9	Mine tailings	24°37′16″ N 101°24′27″ W
	S10	Mine tailings	24°37′15″ N 101°24′45″ W
	S11	Undisturbed soil	24°36′20″ N 101°25′60″ W
	S12	Mine tailings	24°36′49″ N 101°25′37″ W
	S13	Mine tailings	24°36′23″ N 101°25′28″ W
	S14	Mine tailings	24°36′43″ N 101°25′20″ W
	S15	Mine tailings	24°36′57″ N 101°27′26″ W
	S16	Undisturbed soil	24°37′17″ N 101°24′48″ W
	S17	Undisturbed soil	24°36′42″ N 101°24′41″ W
	S18	Undisturbed soil	24°36′46″ N 101°25′28″ W
	S19	Undisturbed soil	24°37′30″ N 101°24′90″ W
	S20	Undisturbed soil	22°46′54″ N 102°36′50″ W
	S21	Mine tailings	24°37′20″ N 101°27′23″ W

**Table 2:** Groups of gamma-emitting radionuclides utilized in this work, according to the classification by Murray et al. [27] and Gil-Pacheco et al. [28]. The shaded groups were not included in this study.

Group	Radionuclide Long Half-Life	Gamma-Emitting Nuclide	Gamma-Ray Energy (keV)	Gamma-Emitting Nuclides Included
A	^238^U	^234^Th ^234m^Pa	63.30 ± 0.02 1001.30 ± 0.02	^238^U, ^234^Th ^234^Pa, ^234m^Pa
B	^230^Th	^230^Th	67.67 ± 0.01	^230^Th
C	^226^Ra ^222^Rn	^226^Ra ^214^Pb ^214^Bi	186.21 ± 0.01 351.932 ± 0.002 609.312 ± 0.007 1120.29 ± 0.01 1764.49 ± 0.01	^226^Ra, ^222^Rn ^214^Pb, ^214^Bi ^210^Tl
D	^210^Pb	^210^Pb	46.539 ± 0.001	^210^Pb
E	^235^U	^235^U	143.767 ± 0.003 163.356 ± 0.003 205.316 ± 0.004	^235^U series
F	^232^Th ^228^Ra	(-) ^228^Ac	(-) 911.196 ± 0.002	^232^Th, ^228^Ra ^228^Ac
G	^228^Th ^220^Rn	^224^Ra ^212^Pb ^208^Tl	(-) 238.632 ± 0.002 583.187 ± 0.002	^228^Th, ^224^Ra ^220^Rn, ^212^Pb ^212^Bi, ^208^Tl

**Table 3:** Results of specific activity concentration (Bq kg^−1^) of zones A and B samples and world average radionuclide content in soil in Bq kg^−1^, UNSCEAR 2000 [5].

	Sample	Group A ^238^U	Group C ^226^Ra	Group F ^232^Th	Group G ^228^Th	^40^K	^137^Cs
**Zone A**	S1	62.81 ± 1.44	165.48 ± 0.98	24.35 ± 0.55	23.94 ± 0.61	967.58 ± 13.11	0.68 ± 0.02
	S2	52.26 ± 1.48	259.98 ± 1.64	40.77 ± 0.92	48.22 ± 1.24	1356.02 ± 18.37	0.63 ± 0.02
	S3	51.22 ± 1.18	171.46 ± 1.05	25.92 ± 0.58	27.49 ± 0.7	1123.12 ± 15.21	0.07 ± 0.01
	S4	80.71 ± 1.49	303.45 ± 1.9	58.93 ± 1.32	69.26 ± 1.79	1348.26 ± 18.26	2.87 ± 0.08
	S5	115.92 ± 2.33	124.26 ± 1.09	15.18 ± 0.49	14.92 ± 0.54	845.92 ± 16.56	0.16 ± 0.01
	S6	86.79 ± 1.92	177.86 ± 1.1	24.92 ± 0.56	29.14 ± 0.75	812.99 ± 11.01	1.89 ± 0.05
	Range	51.22–115.92	124.26–303.45	15.18–58.93	14.92–69.26	812.99–1356.02	0.07–2.87
	Mean	**74.95**	**200.42**	**31.68**	**35.5**	**1075.65**	**1.05**
**Zone B**	S7	103.11 ± 2.10	259.98 ± 2.31	12.44 ± 0.28	9.64 ± 0.24	401.35 ± 5.44	0.58 ± 0.02
	S8	97.91 ± 1.98	259.98 ± 2.13	27.41 ± 0.62	26.69 ± 0.68	949.28 ± 12.86	2.10 ± 0.06
	S9	59.41 ± 1.80	105.76 ± 0.62	9.66 ± 0.22	6.70 ± 0.16	324.17 ± 4.13	2.00 ± 0.06
	S10	60.13 ± 1.54	242.20 ± 1.50	18.22 ± 0.41	13.49 ± 0.33	597.77 ± 8.10	1.07 ± 0.03
	S11	87.11 ± 1.81	315.91 ± 2.05	37.02 ± 0.86	42.80 ± 1.14	1121.25 ± 15.69	0.69 ± 0.02
	S12	120.25 ± 3.72	276.49 ± 1.72	23.97 ± 0.54	22.39 ± 0.57	732.21 ± 9.92	2.09 ± 0.06
	S13	160.18 ± 2.99	292.74 ± 1.82	24.95 ± 0.56	22.94 ± 0.58	851.84 ± 11.54	0.57 ± 0.02
	S14	152.28 ± 1.98	596.26 ± 3.86	15.72 ± 0.35	14.23 ± 0.36	354.92 ± 4.81	1.10 ± 0.03
	S15	106.03 ± 2.09	308.47 ± 1.89	26.24 ± 0.57	27.11 ± 0.67	824.09 ± 10.73	2.12 ± 0.06
	S16	112.86 ± 2.44	324.65 ± 1.63	30.14 ± 0.54	32.92 ± 0.67	1237.67 ± 13.28	0.41 ± 0.01
	S17	95.43 ± 2.05	281.92 ± 2.01	23.19 ± 0.58	22.84 ± 0.65	658.66 ± 10.01	6.35 ± 0.21
	S18	151.63 ± 2.52	503.49 ± 2.61	48.32 ± 0.88	57.07 ± 1.19	929.78 ± 10.20	0.39 ± 0.01
	S19	99.94 ± 1.64	251.54 ± 1.07	43.49 ± 0.67	50.98 ± 0.90	1309.66 ± 12.11	0.14 ± 0.01
	S20	120.99 ± 1.54	190.07 ± 0.79	31.63 ± 0.48	32.23 ± 0.56	1086.02 ± 10.04	2.10 ± 0.04
	S21	82.55 ± 1.18	390.56 ± 2.52	44.97 ± 1.01	53.31 ± 1.37	1347.95 ± 18.26	0.59 ± 0.02
	Range	59.41–160.18	105.76–596.26	9.66–48.32	6.70–57.07	324.17–1347.95	0.14–6.35
	Mean	**107.32**	**306.67**	**27.82**	**29.02**	**883.69**	**1.48**
**World** **average**	Range	16–110	17–60	11–64		140–850	
	Mean	**35**	**35**	**30**		**400**	

**Table 4:** Average specific activity concentration of the radionuclides studied in this work and other parts of the world [4].

	^238^U (Bq kg^−1^)	^226^Ra (Bq kg^−1^)	^232^Th (Bq kg^−1^)	^40^K (Bq kg^−1^)
This work, zone A	75	200	35	1076
This work, zone B	107	307	28	884
Algeria	30	50	25	370
Egypt	37	17	18	320
Costa Rica	46	46	11	140
United states	35	40	35	370
China	33	32	41	440
Hong Kong	84	59	95	530
India	29	29	64	400
Japan	29	33	28	310
Kazakhstan	37	35	60	300
Malaysia	66	67	82	310
Thailand	114	48	51	230
Armenia	46	51	30	360
Syrian Arab Republic	23	20	20	270
Norway	50	50	45	850
Ireland	37	60	26	350
Switzerland	40	40	25	370
Bulgaria	40	45	30	400
Hungary	29	33	28	370
Poland	26	26	21	410
Romania	32	32	38	490
Russian Federation	19	27	30	520
Slovakia	32	32	38	520
Croatia	110	54	45	490
Greece	25	25	21	360
Portugal	49	44	51	840
World average	**40**	**37**	**36**	**369**

**Table 5:** Parameters obtained from the activity concentration of the samples from zones A and B.

	Sample	*Ra*_*eq*_ Bq kg^−1^	$(\dot{D}$) nGy h^−1^	E mSv y^−1^	ELCR mSv	H_ex_ mSv y^−1^	H_in_ mSv y^−1^	R_i_ nGy h^−1^
**Zone A**	S1	275.23	134.69	0.16	0.00065	0.744	1.191	0.76
	S2	422.70	201.28	0.25	0.00100	1.142	1.845	1.18
	S3	295.01	141.70	0.17	0.00070	0.797	1.260	0.82
	S4	491.53	232.01	0.28	0.00115	1.328	2.148	1.37
	S5	211.11	101.85	0.12	0.00050	0.570	0.906	0.59
	S6	276.10	131.13	0.16	0.00065	0.113	0.113	0.77
	Range	(211.11–491.53)	(101.85–232.01)	(0.12–0.28)	(0.0005–0.0012)	(0.11–1.33)	(0.11–2.15)	(0.59–1.37)
	Mean	**328.61**	**157.11**	**0.19**	**0.0008**	**0.78**	**1.24**	**0.92**
**Zone B**	S7	409.54	190.96	0.23	0.00095	1.107	2.082	1.10
	S8	443.37	209.10	0.26	0.00104	1.198	2.093	1.21
	S9	144.54	68.22	0.08	0.00034	0.391	0.676	0.39
	S10	314.28	147.83	0.18	0.00073	0.849	1.504	0.85
	S11	455.18	215.07	0.26	0.00107	1.230	2.084	1.26
	S12	367.14	172.75	0.21	0.00086	0.992	1.739	1.00
	S13	393.86	185.77	0.23	0.00092	1.064	1.855	1.07
	S14	646.07	299.77	0.37	0.00149	1.746	3.358	1.73
	S15	409.45	192.73	0.24	0.00096	1.106	1.940	1.12
	S16	463.06	219.81	0.27	0.00109	1.251	2.129	1.28
	S17	365.80	171.72	0.21	0.00085	0.988	1.750	1.00
	S18	644.18	300.57	0.37	0.00147	1.741	3.101	1.76
	S19	414.57	197.09	0.24	0.00098	1.120	1.800	1.16
	S20	318.92	152.20	0.19	0.00076	0.862	1.375	0.88
	S21	558.67	263.81	0.32	0.00131	1.509	2.565	1.55
	Range	(144.54–646.07)	(68.22–300.57)	(0.08–0.37)	(0.0003–0.0015)	(0.39–1.75)	(0.68–3.36)	(0.39–1.76)
	Mean	**423.24**	**199.16**	**0.24**	**0.001**	**1.14**	**2.00**	**1.16**

**Table 6:** pH and concentration of major elements (%) obtained with XRF belonging to zones A and B.

	Sample ID	pH Water	pH CaCl_2_	Si	S	Fe	Ca	Al	Mg	Na	K	Ti	P
**Zone A**	**S1**	3.4	3.4	23	4.3	3.7	2.6	2.1	0.73	-	0.64	0.06	0.02
	**S2**	8.3	7.9	19	0.32	8.1	0.9	8.6	1.1	0.39	2.2	0.71	0.07
	**S3**	7.5	5.1	21	0.15	3.1	6.3	6.2	0.49	0.70	2.2	0.34	0.29
	**S4**	9.6	9.2	22	0.057	4.6	0.5	9.1	0.95	0.54	2.4	0.46	0.11
	**S5**	4.8	4.5	25	1.3	2.3	1.5	8.0	0.67	0.25	1.5	0.14	0.27
	**S6**	8.3	7.7	11	2.8	17	11	2.9	0.40	0.42	0.52	0.12	-
	**Mean**	**7**	**6.3**	**20.16**	**1.48**	**6.46**	**3.8**	**6.15**	**0.72**	**0.46**	**1.57**	**0.3**	**0.15**
**Zone B**	**S7**	7.2	6.9	6.6	7.4	19	15	1.0	0.20	0.10	0.27	0.05	0.04
	**S8**	7.8	7.4	9.3	3.1	15	20	0.7	0.38	0.07	0.12	0.04	0.03
	**S9**	7.8	7.4	8.7	3.1	15	19	0.7	0.29	-	0.10	0.04	0.02
	**S10**	7.3	7.2	7.6	5.9	21	14	1.1	0.24	-	0.23	0.06	0.05
	**S11**	8.3	7.6	16	4.0	8.0	6.7	5.4	0.7	0.68	1.8	0.27	0.18
	**S12**	6.9	6.7	16	1.7	12	5.2	5.9	0.39	0.27	0.70	0.30	0.22
	**S13**	7.5	7.7	11	1.9	14	18	1.4	0.45	0.23	0.33	0.05	-
	**S14**	7.6	7.3	9.3	0.46	7.8	27	0.4	0.55	-	0.48	0.02	0.01
	**S15**	7.9	7.2	10	0.46	8.6	24	0.6	0.47	-	0.13	0.03	0.01
	**S16**	8.4	8.0	10	0.083	3.4	25	3.5	0.45	0.14	1.6	0.26	-
	**S17**	8.3	7.7	3.2	0.036	1.0	40	1.1	0.75	0.11	0.14	0.08	0.04
	**S18**	8.2	7.8	4.4	0.11	3.0	35	1.3	0.35	0.09	0.39	-	0.08
	**S19**	8.1	7.7	8.8	0.11	3.3	27	2.8	0.55	0.13	0.86	0.21	0.09
	**S20**	8.1	7.6	8.8	0.12	3.1	26	2.6	0.43	0.18	1.0	0.21	0.10
	**S21**	8.3	7.7	22	0.25	5.1	1.0	9.2	1.9	0.22	2.8	0.54	0.08
	**Mean**	**7.84**	**7.46**	**10.11**	**1.91**	**9.28**	**20.19**	**2.51**	**0.54**	**0.2**	**0.73**	**0.15**	**0.07**

**Table 7:** Concentration of HMs obtained by ICP-MS belonging to zones A and B (mg kg-^1^) and total reference concentrations by soil type [38].

	Sample ID	As	Cd	Cr	Ni	Pb	Se	Tl	U	Th
**Zone A**	S1	180.08 ± 0.55	5.20 ± 0.65	24.44 ± 0.21	18.22 ± 0.14	533.74 ± 3.00	2.65 ± 0.06	1.20 ± 0.15	0.72 ± 0.10	0.80 ± 0.12
	S2	169.78 ± 0.43	0.54 ± 0.39	111.61 ± 0.28	41.23 ± 0.08	1465.76 ± 3.20	1.98 ± 0.03	3.20 ± 0.21	1.10 ± 0.07	0.23 ± 0.02
	S3	58.39 ± 0.16	0.15 ± 0.22	43.51 ± 0.07	14.13 ± 0.03	162.92 ± 0.32	2.46 ± 0.02	1.14 ± 0.12	1.39 ± 0.18	1.85 ± 0.20
	S4	60.31 ± 0.28	0.08 ± 0.11	60.31 ± 0.28	24.57 ± 0.05	124.08 ± 0.36	0.55 ± 0.01	0.85 ± 0.10	0.80 ± 0.10	0.17 ± 0.01
	S5	82.27 ± 0.29	0.17 ± 0.24	28.61 ± 0.20	11.71 ± 0.10	101.26 ± 0.74	1.21 ± 0.04	1.40 ± 0.13	1.92 ± 0.25	1.10 ± 0.14
	S6	23.01 ± 0.11	2.58 ± 0.14	149.41 ± 1.3	58.53 ± 0.45	0.23 ± 0.15	1.41 ± 0.02	1.50 ± 0.07	1.46 ± 0.07	1.21 ± 0.007
	Range	23.01–180.08	0.08–5.20	24.44–149.41	11.71–58.53	0.23–1465	0.55–2.65	0.85–3.20	0.72–1.92	0.17–1.85
	Mean	**90.22**	**1.45**	**69.65**	**28.06**	**398**	**1.71**	**1.55**	**1.23**	**0.89**
**Zone B**	S7	517.05 ± 1.8	1.39 ± 1.8	14.12 ± 0.04	21.51 ± 0.03	768.40 ± 2.60	14.58 ± 0.15	0.98 ± 0.12	6.68 ± 1.05	0.28 ± 0.03
	S8	200.37 ± 1.1	0.29 ± 0.17	7.89 ± 0.05	20.97 ± 0.11	87.54 ± 0.71	7.65 ± 0.15	0.29 ± 0.03	5.37 ± 0.71	0.03 ± 0.005
	S9	476.47 ± 2.0	0.35 ± 0.13	7.28 ± 0.03	12.45 ± 0.11	103.14 ± 0.24	5.43 ± 0.11	0.23 ± 0.01	4.31 ± 0.19	0.01 ± 0.002
	S10	457.19 ± 2.0	0.30 ± 0.13	5.98 ± 0.03	13.27 ± 0.05	98.42 ± 0.39	5.64 ± 0.08	0.20 ± 0.01	4.55 ± 0.31	0.003 ± 0.001
	S11	219.73 ± 0.3	0.29 ± 0.16	8.13 ± 0.05	27.27 ± 0.10	123.90 ± 0.51	9.91 ± 0.19	0.36 ± 0.02	5.23 ± 0.45	0.02 ± 0.002
	S12	183.21 ± 0.6	0.18 ± 0.24	14.50 ± 0.06	8.88 ± 0.04	59.44 ± 0.19	5.10 ± 0.06	0.95 ± 0.17	3.62 ± 0.60	3.34 ± 0.67
	S13	135.41 ± 0.7	0.09 ± 0.04	13.12 ± 0.06	9.91 ± 0.06	22.52 ± 0.15	3.36 ± 0.07	0.35 ± 0.01	2.95 ± 0.21	3.50 ± 0.29
	S14	199.33 ± 1.4	0.09 ± 0.04	6.17 ± 0.05	10.98 ± 0.07	50.93 ± 0.46	4.05 ± 0.11	0.26 ± 0.01	3.71 ± 0.30	0.01 ± 0.002
	S15	44.25 ± 0.1	0.39 ± 0.11	1.78 ± 0.01	2.11 ± 0.01	224.19 ± 1.01	1.12 ± 0.04	0.14 ± 0.01	2.43 ± 0.08	0.003 ± 0.001
	S16	340.74 ± 3.7	0.63 ± 0.24	2.77 ± 0.03	1.93 ± 0.02	345.83 ± 4.08	7.22 ± 0.06	0.17 ± 0.01	3.03 ± 0.15	0.003 ± 0.001
	S17	217.77 ± 1.6	6.12 ± 0.42	21.92 ± 0.18	16.37 ± 0.11	4.35 ± 3.58	3.32 ± 0.04	4.85 ± 0.33	3.04 ± 0.23	1.050 ± 0.008
	S18	51.98 ± 0.28	2.37 ± 0.08	7.62 ± 0.03	5.28 ± 0.02	0.83 ± 0.60	1.53 ± 0.03	0.25 ± 0.01	1.05 ± 0.05	0.170 ± 0.001
	S19	182.49 ± 1.3	5.57 ± 0.41	13.09 ± 0.13	13.78 ± 0.14	2.58 ± 2.64	2.70 ± 0.06	0.59 ± 0.04	2.26 ± 0.16	0.390 ± 0.003
	S20	141.25 ± 0.5	4.09 ± 0.29	13.95 ± 0.07	12.36 ± 0.06	1.88 ± 0.84	2.64 ± 0.04	1.26 ± 0.07	1.85 ± 0.13	0.970 ± 0.004
	S21	153.87 ± 1.0	5.36 ± 0.29	13.94 ± 0.08	13.15 ± 0.07	3.23 ± 2.32	2.70 ± 0.03	3.57 ± 0.15	1.85 ± 0.11	0.620 ± 0.004
	Range	44.3–517.7	0.1–6.1	1.7–21.9	1.9–27.3	0.8–768	1.1–14.6	0.1–4.8	1.1–6.7	0.003–3.50
	Mean	**234.7**	**1.8**	**10.2**	**12.7**	**126**	**5.1**	**0.96**	**3.5**	**0.70**
**Agricultural/** **residential/** **commercial use**	Mean	**22**	**37**	**280**	**1600**	**400**	**390**	**5.2**		
**Industrial use**	Mean	**260**	**450**	**510**	**20,000**	**800**	**5100**	**67**		

**Table 8:** Crystalline characterization of zone A and B samples.

	Sample ID	Oxides	Carbonates	Silicates	Hydroxides	Other Compounds
**Zone A**	S1	SiO_2_, Fe_2_O_3_	CaCO_3_	LiAlSiO_4_		CaF_2_
	S2	SiO_2_, Fe_2_O_3_		KAlSi_3_O_8_ Na(AlSi_3_O_8_) Na_0.3_(Al,Mg)_2_Si_4_O_10_(OH)_2_.H_2_O CaAl_2_Si_2_O_8_4H_2_O		
	S3	SiO_2_	CaCO_3_	K(Al,Fe)Si_2_O_8_ Ca_0.88_S_0.12_Al_1.77_Si_2.23_O_8_ KAlSi_3_O_8_ Na_0.3_(Al,Mg)_2_Si_4_O_10_(OH)_2_.H_2_O		
	S4	SiO_2_		(Na_0.98_Ca_0.02_)(Al_1.02_Si_2.98_O_8_) K(Al_4_Si_2_O_9_(OH)_3_) K_4_Al_4_Si_12_O_32_		
	S5	SiO_2_, Fe_2_O_3_	CaCO_3_	KAlSi_3_O_8_ KAl_2_Si_3_AlO_10_(OH)_2_		
	S6	SiO_2_, Fe_2_O_3_	CaCO_3_	Ca_3_Fe_1.88_(SiO_4_)_3_ KAlSi_3_O_8_ Ca_0.88_S_0.12_Al_1.77_Si_2.23_O_8_		CaSO_4_
**Zone B**	S7	SiO_2_, Fe_2_O_3_	CaCO_3_	Ca_3_Fe_1.88_(SiO_4_)_3_ K(Al Fe)Si_2_O_8_		CaSO_4_
	S8	SiO_2_, Fe_2_O_3_	CaCO_3_	Ca_3_Fe_1.88_(SiO_4_)_3_		CaSO_4_
	S9	SiO_2_, Fe_2_O_3_	CaCO_3_	Ca_3_Fe_1.88_(SiO_4_)_3_		CaSO_4_
	S10	SiO_2_, Fe_2_O_3_	CaCO_3_	Ca_3_Fe_1.88_(SiO_4_)_3_		CaSO_4_
	S11	SiO_2_, Fe_2_O_3_	CaCO_3_	K(Al Fe)Si_2_O_8_ Ca_3_Fe_1.88_(SiO_4_)_3_ (Na,Ca)Al(Si,Al)_3_O_8_		CaSO_4_
	S12	SiO_2_, Fe_2_O_3_	CaCO_3_	K(Al Fe)Si_2_O_8_ Ca_3_(Al_1. 3325_Fe_0. 6675_)Si_3_O_12_		CaSO_4_
	S13	SiO_2_, Fe_2_O_3_	CaCO_3_	Ca_3_Fe_1.88_(SiO_4_)_3_ Ca_0.88_S_0.12_Al_1.77_Si_2.23_O_8_		CaSO_4_
	S14	SiO_2_, Fe_2_O_3_	CaCO_3_	Ca_3_Fe_1.88_(SiO_4_)_3_		
	S15	SiO_2_, Fe_2_O_3_	CaCO_3_	Ca_3_Fe_1.88_(SiO_4_)_3_		
	S16	SiO_2_	CaCO_3_	K(Al Fe)Si_2_O_8_ KAl_2_Si_3_AlO_10_(OH)_2_ (Mg Al)5(Si Al)_8_O20(OH)_2_8H_2_O Ca_0.88_S_0.12_Al_1.77_Si_2.23_O_8_ K(AlSi_3_O_8_) Ca_3_Al_2_(SiO_4_)_2_(OH)_4_		
	S17	SiO_2_	CaCO_3_	Ca_3_Al_2_SiO_6_ CaMg_3_(SiO_4_)_3_		
	S18	SiO_2_	CaCO_3_	Ca_3_Al_2_(SiO_4_)_2_(OH)_4_ CaAl_2_Si_2_O_8_ K(AlSi_3_O_8_)	Fe(OH)_3_	
	S19	SiO_2_	CaCO_3_	Ca_2.964_(Al_1.026_Fe_0.974_)Si_2.979_O_11.844_(OH)_0.156_ KAl_4_(Si Al)_8_O_10_(OH)4H_2_O Ca_0.88_S_0.12_Al_1.77_ Si_2.23_O_8_		
	S20	SiO_2_	CaCO_3_	Ca_0.88_S_0.12_Al_1.77_Si_2.23_O_8_ KAl_4_(Si Al)_8_O_10_(OH)4H_2_O Ca_2.964_(Al_1.026_Fe_0.974_)Si_2.979_O_11.844_(OH)_0.156_ Ca_2_Al_2_SiO_6_(OH)_2_		
	S21	SiO_2_		KAl_2_Si_3_AlO_10_(OH)_2_ Al_2_Si_2_O_5_(OH)_4_ CaAl_2_Si_7_O_18_.7.5H_2_O (Mg Fe Al)_6(_Si Al)_4_O_10_(OH)_8_ KAlSi_3_O_8_		

**Table 9:** Enrichment factor (EF) of zones A and B.

	Sample ID	As	Cd	Cr	Ni	Pb	Se	Tl	U	Th
Zone A	S1	12.71	5.51	0.29	0.28	8.07	4.68	1.41	0.28	0.08
	S2	5.47	0.26	0.60	0.29	10.13	1.60	1.72	0.20	0.01
	S3	4.92	0.19	0.61	0.26	2.94	5.18	1.6	0.65	0.22
	S4	1.58	0.07	0.57	0.31	1.51	0.78	0.8	0.25	0.01
	S5	9.34	0.29	0.54	0.29	2.46	3.42	2.66	1.21	0.18
	S6	1.18	1.98	1.28	0.66	0.00	1.81	1.28	0.41	0.09
Zone B	S7	7.94	0.32	0.04	0.07	2.53	5.60	0.25	0.57	0.01
	S8	2.75	0.06	0.02	0.06	0.26	2.63	0.07	0.41	<0.01
	S9	8.3	0.09	0.02	0.05	0.38	2.37	0.07	0.42	<0.01
	S10	7.96	0.08	0.02	0.05	0.37	2.45	0.06	0.44	<0.01
	S11	2.73	0.05	0.02	0.07	0.33	3.08	0.08	0.36	<0.01
	S12	5.98	0.09	0.08	0.06	0.42	4.16	0.52	0.66	0.16
	S13	2.95	0.03	0.05	0.05	0.11	1.83	0.13	0.36	0.11
	S14	3.72	0.03	0.02	0.05	0.20	1.89	0.08	0.38	<0.01
	S15	1.48	0.20	0.01	0.02	1.61	0.94	0.08	0.45	<0.01
	S16	10.35	0.29	0.01	0.01	2.25	5.48	0.09	0.51	<0.01
	S17	16.73	7.05	0.28	0.28	0.07	6.38	6.21	1.30	0.12
	S18	13.58	9.29	0.33	0.30	0.05	9.97	1.08	1.53	0.06
	S19	15.89	7.27	0.19	0.26	0.05	5.88	0.85	1.09	0.05
	S20	11.18	4.86	0.18	0.22	0.03	5.22	1.66	0.81	0.11
	S21	12.96	6.78	0.20	0.24	0.06	5.68	5.01	0.87	0.08

**Table 10:** Geoaccumulation index (I_geo_) in zones A and B.

	Sample ID	As	Cd	Cr	Ni	Pb	Se	Tl	U	Th
**Zone A**	S1	3.00	1.79	−2.47	−2.49	2.35	1.56	−0.17	−2.49	−4.30
	S2	2.92	−1.48	−0.27	−1.31	3.80	1.14	1.24	−1.89	−6.08
	S3	1.38	−3.32	−1.63	−2.85	0.63	1.45	−0.24	−1.55	−3.09
	S4	0.31	−4.17	−1.16	−2.05	0.24	−0.72	−0.67	−2.34	−6.55
	S5	1.87	−3.13	−2.24	−3.12	−0.05	0.42	0.06	−1.08	−3.84
	S6	0.03	0.78	0.15	−0.80	−8.81	0.65	0.15	−1.47	−3.70
**Zone B**	S7	4.52	−0.11	−3.26	−2.25	2.87	4.02	−0.46	0.72	−5.84
	S8	3.15	−2.35	−4.10	−2.28	−0.26	3.09	−2.20	0.41	−9.01
	S9	4.40	−2.10	−4.21	−3.03	−0.03	2.59	−2.55	0.09	−10.61
	S10	4.34	−2.34	−4.50	−2.94	−0.09	2.65	−2.78	0.17	−12.42
	S11	3.29	−2.38	−4.05	−1.90	0.24	3.46	−1.89	0.37	−9.34
	S12	3.03	−3.04	−3.22	−3.52	−0.82	2.50	−0.51	−0.16	−2.24
	S13	2.59	−4.09	−3.36	−3.36	−2.22	1.90	−1.95	−0.46	−2.17
	S14	3.15	−3.99	-4.45	−3.22	−1.04	2.17	−2.38	−0.12	−10.15
	S15	0.98	−1.93	−6.24	−5.60	1.09	0.31	−3.31	−0.74	−13.94
	S16	3.92	−1.26	−5.60	−5.72	1.72	3.00	−2.98	−0.42	−12.59
	S17	3.27	2.03	−2.62	−2.64	−4.59	1.88	1.84	−0.42	−3.91
	S18	1.21	0.66	−4.15	−4.27	−6.99	0.76	−2.44	−1.95	−6.51
	S19	3.02	1.89	−3.37	−2.89	−5.34	1.59	−1.20	−0.84	−5.34
	S20	2.65	1.45	−3.27	−3.05	−5.80	1.55	−0.10	−1.13	−4.02
	S21	2.77	1.84	−3.27	−2.96	−5.02	1.58	1.40	−1.13	−4.66

**Table 11:** Contamination factors (CFs) in zones A and B.

	Sample ID	As	Cd	Cr	Ni	Pb	Se	Tl	U	Th
**Zone A**	S1	12.01	5.2	0.27	0.27	7.62	4.42	1.33	0.27	0.08
	S2	11.32	0.54	1.24	0.61	20.94	3.30	3.55	0.41	0.02
	S3	3.89	0.15	0.48	0.21	2.33	4.10	1.27	0.51	0.18
	S4	1.85	0.08	0.67	0.36	1.77	0.91	0.94	0.30	0.02
	S5	5.48	0.17	0.32	0.17	1.45	2.01	1.56	0.71	0.10
	S6	1.53	2.58	1.66	0.86	<0	2.35	1.67	0.54	0.12
**Zone B**	S7	34.47	1.39	0.16	0.32	10.98	24.30	1.09	2.48	0.03
	S8	13.36	0.29	0.09	0.31	1.25	12.74	0.33	1.99	<0
	S9	31.76	0.35	0.08	0.18	1.47	9.06	0.26	1.60	<0
	S10	30.48	0.30	0.07	0.20	1.41	9.40	0.22	1.68	<0
	S11	14.65	0.29	0.09	0.40	1.77	16.52	0.40	1.94	<0
	S12	12.21	0.18	0.16	0.13	0.85	8.50	1.06	1.34	0.32
	S13	9.03	0.09	0.15	0.15	0.32	5.60	0.39	1.09	0.33
	S14	13.29	0.09	0.07	0.16	0.73	6.75	0.29	1.38	<0
	S15	2.95	0.39	0.02	0.03	3.20	1.87	0.15	0.90	<0
	S16	22.72	0.63	0.03	0.03	4.94	12.03	0.19	1.12	<0
	S17	14.52	6.12	0.24	0.24	0.06	5.54	5.39	1.12	0.10
	S18	3.47	2.37	0.08	0.08	0.01	2.54	0.28	0.39	0.02
	S19	12.17	5.57	0.15	0.2	0.04	4.51	0.65	0.84	0.04
	S20	9.42	4.09	0.15	0.18	0.03	4.40	1.40	0.69	0.09
	S21	10.26	5.36	0.15	0.19	0.05	4.49	3.97	0.68	0.06

## Data Availability

The data that support the findings of this study are available from the corresponding author upon reasonable request.
